# Mesocolic hernia after laparoscopic transverse colectomy: A case report

**DOI:** 10.1016/j.ijscr.2019.11.061

**Published:** 2019-12-06

**Authors:** Ken-ichi Oshiro, Koji Koinuma, Misaki Matsumiya, Mariko Takami, Satoshi Inose, Katsumi Kurihara, Hisanaga Horie, Alan Kawarai Lefor, Naohiro Sata

**Affiliations:** a Department of Surgery, Shin-Oyama City Hospital, 2251-1, Oyama, Tochigi 323-0827, Japan; b Department of Surgery, Jichi Medical University, 3311-1, Shimotsuke, Tochigi 329-0498, Japan

**Keywords:** Internal hernia, Laparoscopic colectomy, Transverse colon cancer, Case report

## Abstract

**Introduction:**

Internal hernias are rare after laparoscopic colorectal resections. We report a patient with an internal hernia through a defect in the transverse mesocolon following laparoscopic resection.

**Presentation of case:**

A 52-year-old male underwent laparoscopic colectomy for transverse colon cancer and had an unremarkable postoperative course. Thirty days postoperatively, he presented to the emergency room with sudden onset abdominal pain and vomiting. Enhanced abdominal computed tomography scan showed strangulated small intestine in the left upper abdomen. An internal hernia through the mesenteric defect created during the recent colon resection was suspected, and emergency laparotomy was performed. One hundred thirty cm of small intestine was found herniated through a mesenteric defect. After repositioning the ischemic-appearing intestine, a 5 cm defect in the transverse mesocolon was found which had not been closed during the previous laparoscopic operation. No intestinal resection was needed, and the mesenteric defect closed with non-absorbable sutures. The post-operative course was unremarkable except for paralytic ileus, which resolved without further intervention.

**Discussion:**

The incidence of internal hernia through a mesenteric defect after laparoscopic colorectal resection is quite low. Therefore, routine closure of the mesenteric defect after laparoscopic colorectal resection is not required. However, a left sided defect in the transverse mesocolon might be at higher risk of causing an internal hernia on anatomic grounds.

**Conclusion:**

We believe that mesenteric defects should be closed after laparoscopic resection of the left side of transverse colon, regardless of their size.

## Highlights

• Internal hernias following laparoscopic colorectal resections are very rare.
• A mesenteric defect of the left transverse colon might be at higher risk of an internal hernia on anatomic grounds.
• We believe that a defect in the left transverse mesocolon require closure regardless of its size.

## 1 Introduction

Laparoscopic resection of colorectal cancer has equivalent surgical outcomes with open surgery [1]. Additional benefits of laparoscopic colorectal resection including less pain, faster recovery of bowel function and decreased length of stay have contributed to this approach becoming widely used [1]. Specific complications unique to laparoscopic surgery have also been reported, including port-site hernias [2] and leg compartment syndrome after prolonged Trendelenburg position in the lithotomy position [3]. Internal hernias are infrequent complications following laparoscopic surgery but their incidence has been increasing with the advent of gastric Roux-en-Y bypass for the treatment of obesity [4]. Since internal hernias after laparoscopic colorectal surgery are rarely reported [[5], [6], [7]], little is known about their details.

We report a patient with a mesenteric hernia following laparoscopic colectomy, with a review of the literature.

This work is reported in accordance with SCARE criteria [8].

## 2 Presentation of case

**Fig. 1:**
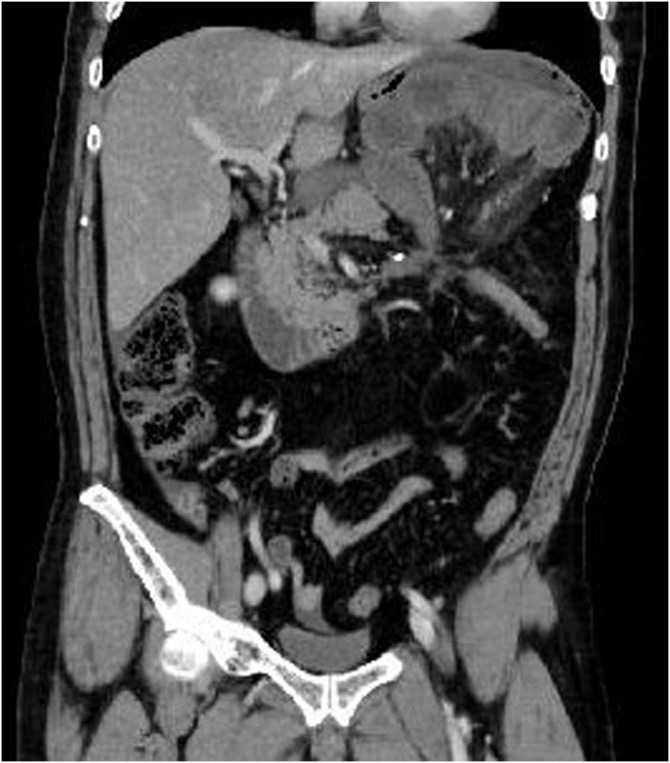
Abdominal CT scan with enhancement shows strangulated small intestine in the left upper abdomen. The strangulated intestine is superior to the transverse mesocolon.

**Fig. 2:**
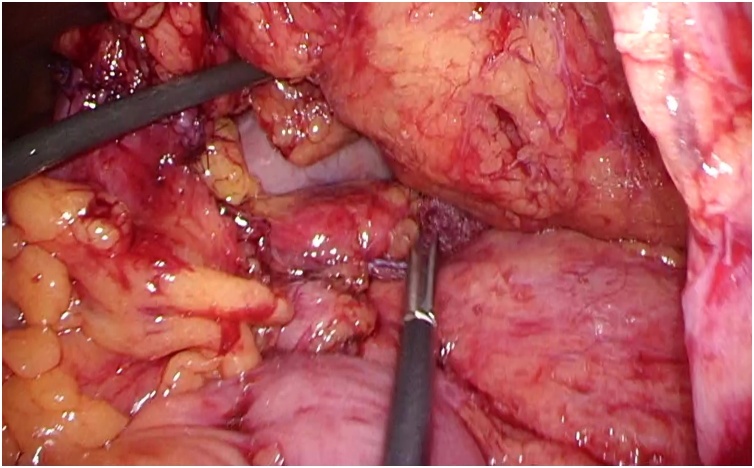
A mesenteric defect was not routinely closed during laparoscopic colectomy.

**Fig. 3:**
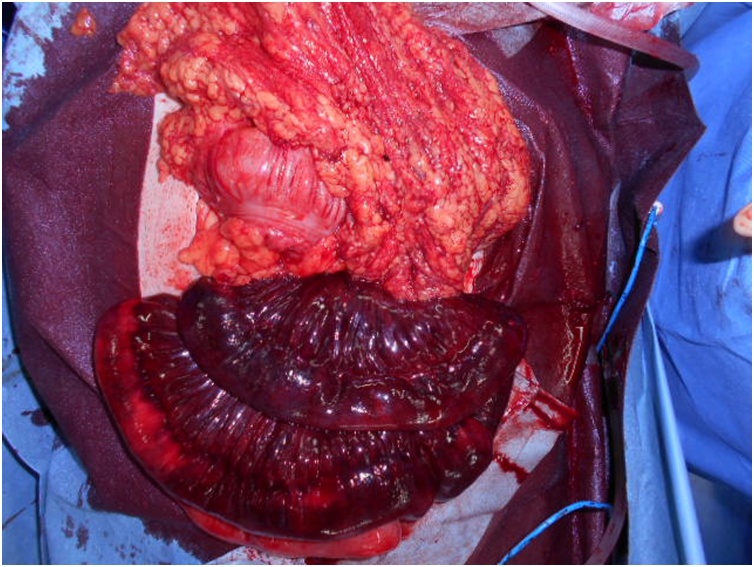
One hundred thirty cm of small intestine herniated through the mesenteric defect and was dark and ischemic.

**Fig. 4:**
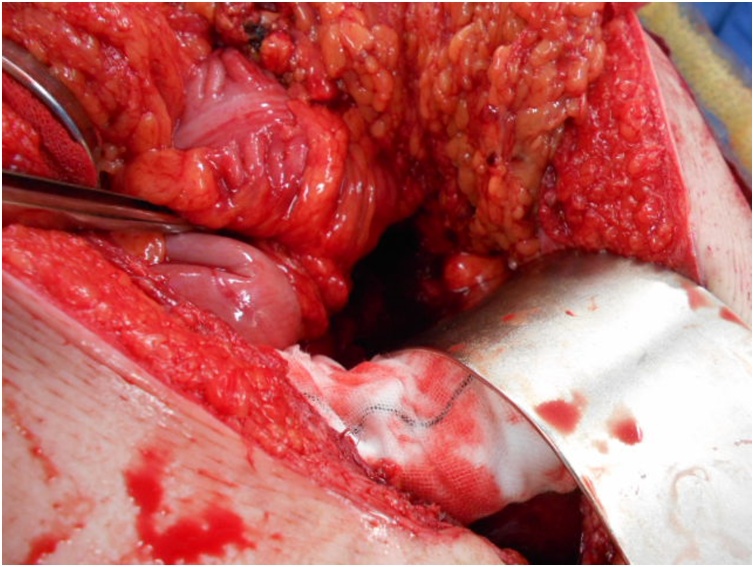
A 5 cm defect in the transverse mesocolon was observed, after repositioning the incarcerated intestine.

A 52-year-old male underwent laparoscopic left hemicolectomy for transverse colon cancer. Mesocolic excision with central ligation of the left branch of the middle colic artery was performed. The post-operative course was uneventful and he was discharged home on the eighth post-operative day. The final pathological diagnosis was pT1, pN0 with TNM stage I. Thirty days postoperatively, he developed acute abdominal pain with vomiting and visited the emergency room. His vital signs were BP 200/110 mmHg, HR 70/min and RR 22/min. Abdominal tenderness with peritoneal signs were observed in the upper abdomen. Serum lactate (4.0 mmol/L) was elevated and other laboratory data were normal. Abdominal computed tomography scan with enhancement showed strangulated small intestine in the left upper abdomen (Fig. 1). The strangulated intestine was superior to the transverse mesocolon, and a small amount of ascites was in the pelvis. We suspected an internal hernia through the mesenteric defect from the previous colon resection. We previously did not routinely close the mesenteric defect during laparoscopic colectomy but packed it with the greater omentum (Fig. 2). Emergency laparotomy was performed. As suspected, 130 cm of small intestine had herniated through the mesenteric defect and was dark and ischemic (Fig. 3). After repositioning the intestine, a 5 cm defect in the transverse mesocolon was observed which had not been closed during the previous laparoscopic operation (Fig. 4). No other adhesions were observed in the abdomen. The blood flow to the intestine gradually recovered, and no intestinal resection was needed. The mesenteric defect was closed with non-absorbable sutures. The post-operative course was unremarkable except for paralytic ileus, which resolved with no specific treatment. There is no evidence of cancer or internal hernia recurrence one year after the first operation.

## 3 Discussion

Internal hernia following laparoscopic colectomy is a rare complication. While it is something that is often discussed, there are few case reports of this complication [[5], [6], [7],^9^,^10^]. Recent reviews suggest that the incidence of symptomatic internal hernias after laparoscopic colorectal resection is from 0.39 to 0.65 % [6,7]. However, these studies have limitations including: (i) small sample size of each study which makes it more likely to overestimate the actual incidence of internal hernias, (ii) heterogeneity of surgical procedures, each study includes more left-sided colorectal resections and fewer transverse colectomies, making the occurrence rate by operation type unclear, (iii) some studies include patients with less than one year follow-up, although internal hernia may develop after one year. In summary, the true incidence is difficult to assess.

To the best of our knowledge, there are only 2 reports of internal hernia through defects in the transverse mesocolon after laparoscopic colon resection [9,10]. This may be due to fewer laparoscopic transverse colon resections, rather than a lower risk of internal hernia in this part of the colon. We believe that this location of transverse to descending colon resection may be at higher risk for internal hernia and clinically important for the following reasons. First, the ligament of Treitz is in the root of the transverse mesocolon and naturally maintains the proximal jejunum close to the posterior abdominal wall. A defect in the mesentery around the ligament of Treitz would lead the jejunum to slide toward the left paracolic gutter [5]. Second, in laparoscopic surgery, there is an increased risk of internal hernia because fewer adhesions are created than after open surgery. There are many reported cases of internal hernias after laparoscopic Roux-en-Y gastric bypass surgery for obesity [4]. Since the peritoneal surface of the mesentery is non-adhesive, small bowel can easily pass through the narrow space between the overlapping mesenteries of Roux-en-Y reconstruction. Anatomically, the transverse colon is not fixed to the retroperitoneum. The left side of the transverse mesocolon is covered with peritoneum on both the superior and inferior sides, which prevent the mesocolon from post-operative adhesions to the retroperitoneum. The other parts of the mesocolon have a raw surface on the side of the retroperitoneum. After dissection, the mesocolon is likely to adhere again to the retroperitoneum. Third, with a mesenteric defect in this area, the most proximal small intestine is likely to be incarcerated. Proximal small bowel obstruction may cause severe gastrointestinal symptoms with subsequent dehydration and metabolic alkalosis [11].

There is no consensus about the need for closure of mesenteric defects following laparoscopic colectomy. Most laparoscopic colorectal surgeons have been aware of the potential risk of this rare complication, however many leave the mesenteric defect open. The reasons may be that closure of mesenteric defects during laparoscopic surgery is time-consuming, technically challenging and may injure the mesenteric vessels [10], as well as few existing case reports of post-operative internal hernias. Hosono et al. recommended that the presence of a narrow defect, less than 5 cm, might increase the risk of developing a symptomatic internal hernia and require complete closure, whereas a large mesenteric defect need not be closed [9]. In the present patient, the mesenteric defect after laparoscopic colonic resection was around 10 cm (Fig. 1), and the hernia orifice at the second operation was about 5 cm. The mesenteric defect would have been partially closed by adhesion, leading to being at increased risk for developing an internal hernia. We routinely fill the mesenteric defect with the greater omentum to prevent incarceration of the small bowel in the final stage of the laparoscopic procedure. The utility of this procedure was introduced previously [10], however it was not effective in the present patient.

## 4 Conclusion

The incidence of internal hernia through a mesenteric defect after laparoscopic colorectal resection is quite low. Therefore, some have advocated that routine closure of the mesenteric defect after laparoscopic colorectal resection is not required. However, we believe that a defect in the left side of the transverse mesocolon may be at a higher risk and require closure regardless of its size.

## Declaration of Competing Interest

All authors have no conflicts of interest.

## Funding

This work did not receive any specific grant from funding agencies in the public, commercial, or not-for-profit sectors.

## Ethical approval

This is a case report and it didn’t require ethical approval from ethics committee according to our institution.

## Consent

Witten consent was obtained from the patient for publication of this case reports and any accompanying images.

## Author contribution

Oshiro K and Koinuma K; Conception of study, acquisition, analysis and interpretation of data.

KoinumaK; Drafting the article.

Matsumiya M, Takami M, Inose S, Kurihara K; Management of case.

Koinuma K, Horie H, Lefor A, Sata N; Critical revision of article and final approval of the version to be submitted.

## Registration of research studies

This is a case report study.

## Guarantor

Koji Koinuma.

## Provenance and peer review

Not commissioned, externally peer-reviewed.
